# A Metagenomics Approach to Frailty in Patients With Cirrhosis Undergoing a Multifactorial Intervention

**DOI:** 10.1111/liv.70418

**Published:** 2025-11-01

**Authors:** Sara Vega‐Abellaneda, Eva Román, Zaida Soler, Mª. Àngels Ortiz, Giacomo Rossi, Lucia Biagini, Elisabet Sánchez, Marc Pons‐Tarin, Luca Laghi, Carlo Mengucci, Naujot Kaur, Maria Poca, Berta Cuyàs, Gerard Serrano‐Gomez, Edilmar Alvarado, Chaysavanh Manichanh, German Soriano

**Affiliations:** ^1^ Vall d'Hebron Institut de Recerca (VHIR) Barcelona Spain; ^2^ Hospital de la Santa Creu i Sant Pau Barcelona Spain; ^3^ CIBERehd, Instituto de Salud Carlos III Madrid Spain; ^4^ University Nursing School EUI Sant Pau Barcelona Spain; ^5^ Universitat Autònoma de Barcelona Barcelona Spain; ^6^ Institut de Recerca Sant Pau (IR SANT PAU) Barcelona Spain; ^7^ University of Camerino Camerino Italy; ^8^ University of Bologna Italy

**Keywords:** cirrhosis, exercise, frailty, microbiota, probiotics

## Abstract

The relationship between frailty and gut microbiota has not been previously addressed in patients with cirrhosis. We studied by metagenomic shotgun sequencing the faecal microbiota composition associated with frailty in 29 patients with cirrhosis from a previous study (Román, Hepatol Commun 2024). Frail and prefrail patients were randomised to a multifactorial intervention (home exercise, branched‐chain amino acids and a multistrain probiotic) or control for 12 months. We observed a positive correlation between the abundance of *Rothia dentocariosa* and the Liver frailty index (LFI), and between *Bacteroides faecis* and gait speed. After the multifactorial intervention, LFI improved and the main changes in the microbiota composition were a decrease in the abundance of *Akkermansia muciniphila* , and an increase in *Streptococcus thermophilus* , *Lactobacillus acidophilus* and several species of *Bifidobacterium*. We conclude that frailty in patients with cirrhosis was associated with a distinct microbiome signature. After a long‐term multifactorial intervention, frailty improved in parallel with changes in microbiome composition.

**Trial Registration:** ClinicalTrials.gov identifier: NCT04243148.

**Abbreviations**
BCAA: branched‐chain amino acid
ccK18: caspase‐cleaved keratin 18
FDR: False discovery rate
LFI: Liver frailty index
MELD: Model for end‐stage liver disease
mtDNA: mitochondrial DNA

## 1 Introduction

In patients with cirrhosis, and independently of the degree of liver failure, frailty is a relevant prognostic factor, associated with mortality, the need for healthcare services, and worsening of health‐related quality of life [1].

In view of its clinical impact, frailty has been identified as a therapeutic target, and several strategies have been proposed for its improvement [1]. Exercise, nutritional interventions, branched‐chain amino acid (BCAA) supplements, testosterone supplementation in men, and probiotics [1, 2] are some of the interventions that have been explored to ameliorate frailty in cirrhosis.

In a recent randomised clinical trial, we observed that a multifactorial intervention combining home‐based exercise, BCAA supplementation and a multistrain probiotic improved frailty in patients with cirrhosis, while also reducing emergency room visits and falls [2]. These beneficial effects were associated with an improvement in biomarkers of inflammation, bacterial translocation, mitochondrial dysfunction, and amino acid and short‐chain fatty acid metabolism [3].

To our knowledge, the potential link between frailty and gut microbiota has not been previously addressed in patients with cirrhosis. To further explore the mechanisms associated with frailty we aimed to analyse the relationship between faecal microbiota composition and frailty, and also the changes in the microbiota following the multifactorial intervention in patients with cirrhosis.

## 2 Patients and Methods

**FIGURE 1:**
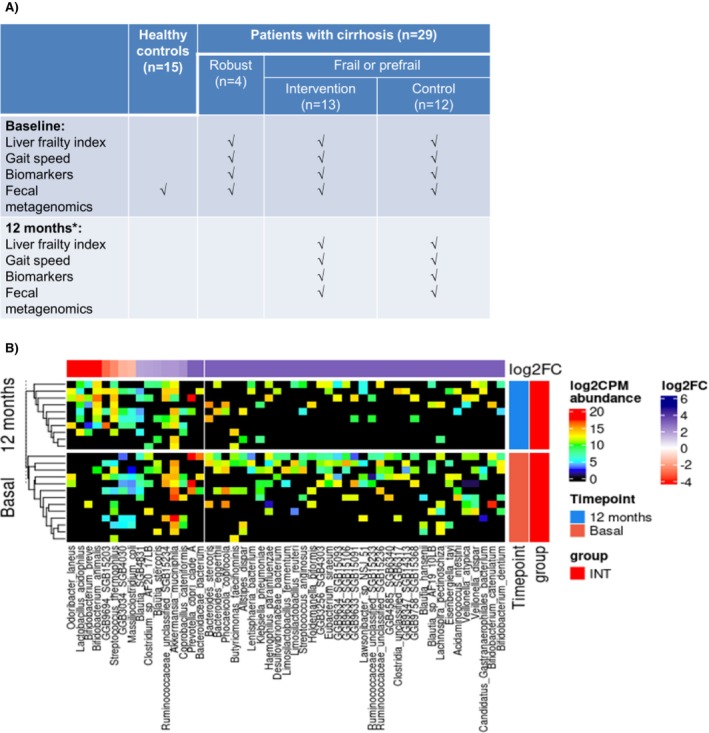
(A) Design of the study; (B) Differentially abundant species between baseline and 12 months in the intervention group with FDR < 0.085. *Ten patients in the intervention group and eight patients in the control group reached the 12 month follow‐up.

We analysed the faecal samples from 29 outpatients with cirrhosis who were part of our previous study in which frail and prefrail patients were randomised to a multifactorial intervention consisting of home‐based exercise, BCAA supplementation and a multistrain probiotic, or control for 12 months [2, 3]. At baseline and at 12 months, frailty was assessed by the Liver frailty index (LFI) and gait speed. Additionally, we analysed a battery of biomarkers in serum and urine [3], and faecal samples were collected for metagenomic shotgun sequencing analysis (Supporting Information). A control group of 15 age and gender‐matched healthy individuals was also included (Figure 1A).

The study conformed to the Guidelines of the Declaration of Helsinki and Istanbul, followed the good clinical practice recommendations and was approved on 23 August 2019 by the ethical committee at our centre (Comitè d'Ètica d'Investigació amb Medicaments), approval number IIBSP‐FRA‐2019‐36, 19/212. All patients gave written informed consent after receiving the appropriate information. The study was registered on ClinicalTrials NCT04243148.

## 3 Results

The 29 outpatients included in the study were 38% women and had (mean ± SD) 64.6 ± 9.1 years of age. MELD score was 8.6 ± 3.2, 23 of the 29 patients had alcohol‐related liver disease, 23 had previous decompensations, and their LFI was 3.9 ± 0.4. In the faecal microbiota analysis, patients with cirrhosis showed an alpha diversity lower than controls (Chao 1, *p* = 0.01). Key differences between patients with cirrhosis and controls included an increased abundance of *Anaerotruncus massiliensis*, *Enterocloster bolteae*, *Streptococcus parasanguinis* and 3 different *Veillonella* species in the cirrhosis group. *Ruminococcus callidus* , an unclassified Ruminococcaceae, *Alistipes putredinis* and an unclassified Clostridia were more abundant in healthy controls. In patients with cirrhosis, microbial diversity did not correlate with the LFI. We observed a positive correlation between the abundance of *Rothia dentocariosa* and LFI (*r* = 0.57, *p* < 0.001), and between *Bacteroides faecis* and gait speed (*r* = 0.60, *p* < 0.001). *Streptococcus thermophilus* was negatively correlated with IL‐6 (r = −0.63, *p* < 0.001), while *Lactobacillus acidophilus* showed a positive correlation with mtDNA (*r* = 0.62, *p* < 0.001). Additionally, the abundances of *Veillonella parvula* and *Veillonella dispar* were positively correlated with ccK18 (*r* = 0.74, *p* < 0.001, and *r* = 0.75, *p* < 0.001, respectively).

In the 10 patients from the multifactorial intervention group that reached the 12 month evaluation, both LFI and gait speed improved (3.31 ± 0.40 vs. 4.01 ± 0.41 at baseline, *p* = 0.005; 1.00 ± 0.09 vs. 0.86 ± 0.18 m/s at baseline, *p* = 0.03, respectively). The most notable changes in the microbiome composition were an increased abundance of *Streptococcus thermophilus* , *Lactobacillus acidophilus* and several species of *Bifidobacterium* after the intervention. Conversely, there was a decrease in *Akkermansia muciniphila* and *Ruminococcus gnavus*, and *Lachnospira pectinoschiza* , *Bifidobacterium catenulatum* , *Bifidobacterium dentium* and *Eubacterium siraeum* disappeared (Figure 1B). No significant changes were observed in the control group. The functional analysis revealed no statistically significant associations or changes related to microbial pathways.

## 4 Discussion

The main finding of this study was an association between frailty and the abundance of specific bacterial species in the microbiota of patients with cirrhosis. To our knowledge, the direct relationship between gut microbiota composition and frailty in cirrhosis has not been studied previously.

Faecal microbial diversity was lower in patients with cirrhosis than in healthy controls, as has been previously reported [4]. Notably, the increase in *Streptococcus parasanguinis*, a bacterium from the oral microbiome [4], and the decrease in Ruminococcaceae, *Alistipes* and *Clostridium* have also been previously shown in cirrhosis [4, 5, 6]. An interesting finding was the increase in *Anaerotruncus massiliensis*, a mucin‐degrading bacterium [7] that may contribute to the impairment of the gut barrier in cirrhosis. The abundance of two *Veillonella* species correlated with ccK18, an index of liver damage. This finding is in line with previous reports showing a relationship between increased *Veillonella* abundance and prognosis in several liver diseases [8].

Regarding frailty, we found a positive correlation between LFI and *Rothia dentocariosa* while gait speed was more impaired in patients with less abundance of *Bacteroides faecis*. These findings align with previous research linking frailty to increased *Rothia* abundance in patients with chronic kidney disease [9] and reduced gait speed to decreased *Bacteroides* in sedentary women [10].

Following the multifactorial intervention, in parallel with frailty improvement we observed changes in the microbiota composition. These changes could be related to the improvement of frailty itself and/or to the combined effect of the components of the multifactorial intervention, as all three components (probiotics, exercise and BCAA) can modulate gut microbiota [11, 12, 13]. The increase in *Streptococcus thermophilus* and *Lactobacillus acidophilus* at the end of the study was to be expected because both were components of the multistrain probiotic administered to patients. In addition, an increase in the abundance of *Lactobacillus* was previously observed in pigs receiving the BCAA leucine [13]. Interestingly, *Streptococcus thermophilus* correlated negatively with serum IL‐6, while *Lactobacillus acidophilus* correlated positively with mtDNA, suggesting these bacteria could have contributed to reducing inflammation and improving mitochondrial function after the intervention [3, 14]. It is of note that in a rat model, *Streptococcus thermophilus* has demonstrated beneficial effects on sarcopenia [15].

Other significant post‐intervention changes were decreases in the abundance of *Akkermansia muciniphila* and *Eubacterium siraeum* . While *Akkermansia* has been reported to be reduced in patients with cirrhosis and sarcopenia [16], it has been found to be increased in frail elderly people [17]. Although the abundance of *Akkermansia* is generally considered beneficial, it has also been associated with Parkinson's disease, multiple sclerosis [18], and mortality in patients admitted to an intensive care unit [19]. Regarding *Eubacterium siraeum* , it has been associated with intestinal barrier dysfunction [20]. Lastly, the lack of statistically significant changes or associations in the microbial functional analysis may be due to the limited sample size, thus highlighting the need for further investigation in larger cohorts.

Within the limitations of the sample size, we conclude that frailty in cirrhosis was associated with a distinct microbiome signature that correlated with biomarkers of inflammation, mitochondrial dysfunction and liver damage. After a long‐term multifactorial intervention, frailty improved in parallel with changes in the microbiome composition. These results provide valuable insights into potential therapeutic targets to improve frailty and its consequences in patients with cirrhosis.

## Author Contributions

Eva Román, Chaysavanh Manichanh and German Soriano: conception and design of the study; Eva Román, Naujot Kaur, Maria Poca, Berta Cuyàs, Edilmar Alvarado and German Soriano selected and controlled participants and collected clinical data; Eva Román and Elisabet Sánchez collected and processed blood and faecal samples; Sara Vega‐Abellaneda, Marc Pons‐Tarin, Zaida Soler and Gerard Serrano‐Gomez performed metagenomic analyses; Mª Àngels Ortiz, Giacomo Rossi, Lucia Biagini, Luca Laghi and Carlo Mengucci performed biomarker determinations; Eva Román, Chaysavanh Manichanh and German Soriano wrote the article. All authors revised the draft and approved the final version of the article.

## Ethics Statement

The study conformed to the Guidelines of the Declaration of Helsinki and Istanbul, followed the good clinical practice recommendations and was approved on 23 August 2019 by the ethical committee at our centre (Comitè d'Ètica d'Investigació amb Medicaments), approval number IIBSP‐FRA‐2019‐36, 19/212. All patients gave written informed consent after receiving the appropriate information.

## Conflicts of Interest

This study was supported by grants from the Fondo de Investigación Sanitaria (FIS), Instituto de Salud Carlos III, Madrid, Spain, co‐funded by the European Union, Fondo Europeo de Desarrollo Regional (FEDER) “Una manera de hacer Europa” (PI19/00275), and Col·legi Oficial d'Infermeres i Infermers de Barcelona (COIB), Spain (PR‐455/2020). Mendes SA (Lugano, Switzerland) partially funded the study and provided the probiotics, and Infisport (Castellón de la Plana, Spain) supplied the branched‐chain amino acids supplements at 50% of the cost.

## Supporting information


**Data S1:** German Soriano.R1.

## Data Availability

The data that support the findings of this study are available on request from the corresponding author. The data are not publicly available due to privacy or ethical restrictions.

## References

[1] P. Tandon , A. J. Montano‐Loza , J. C. Lai , S. Dasarathy , and M. Merli , “Sarcopenia and Frailty in Decompensated Cirrhosis,” Journal of Hepatology 75, no. Suppl 1 (2021): S147–S162. 34039486 10.1016/j.jhep.2021.01.025 PMC9125684

[2] E. Román , N. Kaür , E. Sánchez , et al., “Home Exercise, Branched‐Chain Amino Acids, and Probiotics Improve Frailty in Cirrhosis: A Randomized Clinical Trial,” Hepatology Communications 8 (2024): e0443. 38701490 10.1097/HC9.0000000000000443 PMC11073778

[3] L. Laghi , M. À. Ortiz , G. Rossi , et al., “Biomarkers of Frailty in Patients With Advanced Chronic Liver Disease Undergoing a Multifactorial Intervention Consisting of Home Exercise, Branched‐Chain Amino Acids, and Probiotics,” Biomolecules 14 (2024): 1410. 39595586 10.3390/biom14111410 PMC11592179

[4] C. Solé , S. Guilly , K. da Silva , et al., “Alterations in Gut Microbiome in Cirrhosis as Assessed by Quantitative Metagenomics: Relationship With Acute‐On‐Chronic Liver Failure and Prognosis,” Gastroenterology 160 (2021): 206–218. 32941879 10.1053/j.gastro.2020.08.054

[5] J. S. Bajaj , N. S. Betrapally , P. B. Hylemon , et al., “Salivary Microbiota Reflects Changes in Gut Microbiota in Cirrhosis With Hepatic Encephalopathy,” Hepatology 62 (2015): 1260–1271. 25820757 10.1002/hep.27819 PMC4587995

[6] A. Santiago , M. Pozuelo , M. Poca , et al., “Alteration of the Serum Microbiome Composition in Cirrhotic Patients With Ascites,” Scientific Reports 6 (2016): 25001. 27112233 10.1038/srep25001 PMC4845009

[7] S. Raimondi , E. Musmeci , F. Candeliere , A. Amaretti , and M. Rossi , “Identification of Mucin Degraders of the Human Gut Microbiota,” Scientific Reports 11 (2021): 11094. 34045537 10.1038/s41598-021-90553-4 PMC8159939

[8] K. Yamamoto , T. Honda , Y. Inukai , et al., “Identification of the Microbiome Associated With Prognosis in Patients With Chronic Liver Disease,” Microorganisms 12 (2024): 610. 38543661 10.3390/microorganisms12030610 PMC10974311

[9] E. Margiotta , L. Caldiroli , M. L. Callegari , et al., “Association of Sarcopenia and Gut Microbiota Composition in Older Patients With Advanced Chronic Kidney Disease, Investigation of the Interactions With Uremic Toxins, Inflammation and Oxidative Stress,” Toxins (Basel) 13 (2021): 472. 34357944 10.3390/toxins13070472 PMC8309956

[10] D. A. Piggott and S. Tuddenham , “The Gut Microbiome and Frailty,” Translational Research 221 (2020): 23–43. 32360945 10.1016/j.trsl.2020.03.012 PMC8487348

[11] X. Xu , C. Zhang , G. Tang , N. Wang , and Y. Feng , “Updated Insights Into Probiotics and Hepatobiliary Diseases,” Biomedicine 12 (2024): 515. 10.3390/biomedicines12030515 PMC10968574 38540128

[12] J. Álvarez‐Herms and A. Odriozola , “Microbiome and Physical Activity,” Advances in Genetics 111 (2024): 409–450. 38908903 10.1016/bs.adgen.2024.01.002

[13] M. Beaumont , E. Roura , W. Lambert , C. Turni , J. Michiels , and T. Chalvon‐Demersay , “Selective Nourishing of Gut Microbiota With Amino Acids: A Novel Prebiotic Approach?,” Frontiers in Nutrition 9 (2022): 1066898. 36601082 10.3389/fnut.2022.1066898 PMC9806265

[14] F. Han , G. Wu , Y. Zhang , et al., “ *Streptococcus thermophilus* Attenuates Inflammation in Septic Mice Mediated by Gut Microbiota,” Frontiers in Microbiology 11 (2020): 598010. 33384671 10.3389/fmicb.2020.598010 PMC7769777

[15] I. Savary‐Auzeloux , M. Jarzaguet , C. Migné , et al., “Anti‐Inflammatory *Streptococcus thermophilus* CNRZ160 Limits Sarcopenia Induced by Low‐Grade Inflammation in Older Adult Rats,” Frontiers in Nutrition 9 (2022): 986542. 36245508 10.3389/fnut.2022.986542 PMC9559730

[16] F. R. Ponziani , A. Picca , E. Marzetti , et al., “Characterization of the Gut‐Liver‐Muscle Axis in Cirrhotic Patients With Sarcopenia,” Liver International 41 (2021): 1320–1334. 33713524 10.1111/liv.14876

[17] Y. Xu , Y. Wang , H. Li , et al., “Altered Fecal Microbiota Composition in Older Adults With Frailty,” Frontiers in Cellular and Infection Microbiology 11 (2021): 696186. 34485176 10.3389/fcimb.2021.696186 PMC8415883

[18] V. Chiantera , A. S. Laganà , S. Basciani , M. Nordio , and M. Bizzarri , “A Critical Perspective on the Supplementation of *Akkermansia muciniphila*: Benefits and Harms,” Life 13 (2023): 1247. 37374030 10.3390/life13061247 PMC10301191

[19] R. Prevel , R. Enaud , A. Orieux , et al., “Gut Bacteriobiota and Mycobiota Are Both Associated With Day‐28 Mortality Among Critically Ill Patients,” Critical Care 26 (2022): 105. 35418098 10.1186/s13054-022-03980-8 PMC9007252

[20] F. Yang , H. Chen , Y. Gao , et al., “Gut Microbiota‐Derived Short‐Chain Fatty Acids and Hypertension: Mechanism and Treatment,” Biomedicine & Pharmacotherapy 130 (2020): 110503. 34321175 10.1016/j.biopha.2020.110503

