# Supplementary material for: A Metagenomics Approach to Frailty in Patients With Cirrhosis Undergoing a Multifactorial Intervention
Source: Liver Int. 2025 Nov 1;45(12):e70418. doi: 10.1111/liv.70418 (PMC12579327; doi:10.1111/liv.70418)
Supplement: Supplementary file 1 — Data S1: German Soriano.R1. [file LIV-45-0-s001.doc]

**SUPPLEMENTARY MATERIAL**

**Metagenomics shotgun sequencing**

A total of 58 fecal samples were collected (43 from patients with cirrhosis and 15 from controls). Illumina NovaSeq6000 was used for pair-end 150bp sequencing. The KneadData v0.7.4 pipeline (https://huttenhower.sph.harvard.edu/kneaddata) was used to perform quality control and decontamination, resulting in an average of 39335661 reads per sample. MetaPhlAn v4.0 (1) and HUMAnN v3.6 (2) tools were used for the taxonomic and functional microbiome profiling, respectively, from the metagenomic shotgun sequencing data. The R software (R Core Team, Version 4.3.1, 2023) was used for metagenomics analysis.

Alpha diversity

Alpha-diversity was analysed using Chao1 and Shannon indices computed with the phyloseq R package.

Effect of clinical variables and biomarkers

To explore the effect size of clinical variables (LFI and gait speed) and biomarkers on the microbiome, we used the Adonis2 function from vegan R package. We used Student t-test test to compare alpha diversity between patients with cirrhosis and healthy controls.

Differential abundance analysis

Taxonomic differential abundance analysis was performed using Analysis of Compositions of Microbiomes with Bias Correction (ANCOM-BC) (3). Similarly, pathway differential abundance analysis was performed using linear models with TPM normalized pathway abundances. These analyses were done to find the differences in the abundance of each taxon between patients with cirrhosis and healthy controls, setting the group as fixed effect. Additionally, to find differences within the control group between baseline and 12 months, and within the intervention group between baseline and 12 months, analyses with ANCOM-BC were performed using timepoint as the only variable.

Spearman's correlation analysis

We performed Spearman's correlation analysis between the CPM-normalized abundance of all the identified species and the clinical variables, as well as between the pathway abundance and the clinical variables. All the correlations were computed and then p-values were FDR corrected.

Supplementary references

1. Blanco-Míguez A, Beghini F, Cumbo F, et al. Extending and improving metagenomic taxonomic profiling with uncharacterized species using MetaPhlAn 4. Nat Biotechnol 2023;41:1633-1644.
2. Beghini F, McIver LJ, Blanco-Míguez A, et al. Integrating taxonomic, functional, and strain-level profiling of diverse microbial communities with bioBakery 3. Elife 2021;10:e65088.
3. Lin H, Peddada SD. Multigroup analysis of compositions of microbiomes with covariate adjustments and repeated measures. Nat Methods 2024;21:83-91.
